# Electron and heat transport in porphyrin-based single-molecule transistors with electro-burnt graphene electrodes

**DOI:** 10.3762/bjnano.6.146

**Published:** 2015-06-26

**Authors:** Hatef Sadeghi, Sara Sangtarash, Colin J Lambert

**Affiliations:** 1 Quantum Technology Centre, Physics Department, Lancaster University, Lancaster, LA1 4YB, UK

**Keywords:** electro-burnt graphene electrodes, nanoelectronics, porphyrin, single-molecule transistor

## Abstract

We have studied the charge and thermal transport properties of a porphyrin-based single-molecule transistor with electro-burnt graphene electrodes (EBG) using the nonequilibrium Green’s function method and density functional theory. The porphyrin-based molecule is bound to the EBG electrodes by planar aromatic anchor groups. Due to the efficient π–π overlap between the anchor groups and graphene and the location of frontier orbitals relative to the EBG Fermi energy, we predict HOMO-dominated transport. An on–off ratio as high as 150 is predicted for the device, which could be utilized with small gate voltages in the range of ±0.1 V. A positive thermopower of +280 μV/K is predicted for the device at the theoretical Fermi energy. The sign of the thermopower could be changed by tuning the Fermi energy. By gating the junction and changing the Fermi energy by +10 meV, this can be further enhanced to +475 μV/K. Although the electrodes and molecule are symmetric, the junction itself can be asymmetric due to different binding configurations at the electrodes. This can lead to rectification in the current–voltage characteristic of the junction.

## Introduction

Transistors are the fundamental building blocks of modern electronic devices and are used to amplify or switch electronic signals. The most common transistors contain three terminals, two of which carry a current from the source to the drain and the third (gate or base) controls the current through the transport channel to either amplify the input current in bipolar junction transistors (BJT) or switch the voltage in field effect transistors (FET) [1–2]. Recently, the idea of two-terminal molecular-scale transistors has been proposed in which a molecule forms the conducting channel of the transistor [3].

According to Moore’s law [4], the number of transistors in an integrated circuit should double approximately every two years. Since 1974, when the idea of a single-molecule rectifier was proposed by Ratner and Aviram [5], many attempts to realize FETs have failed to deliver the room-temperature reliability, reproducibility and stability required by the electronics industry [6–7]. Transport in single-molecule FETs is mostly dominated by the contacts between the electrodes and molecules, which is an obstacle to achieving reproducibility and stability. Furthermore, in laboratory-based conductance measurements (using, for example, mechanically-controlled break junctions), gold is widely employed [8] as the electrode material in order to avoid oxidation and degradation of the electrodes and because of its high atomic mobility. However, gold nanoelectrodes are unstable at room temperature [6].

Recently, an alternative strategy for the fabrication of stable electrodes with nanometre separation has been proposed using the sp^2^-bonded two-dimensional carbon-based material, graphene [9]. In addition to the excellent stability and conductivity of graphene even at high temperatures, the significant advantage of graphene electrodes with respect to single-molecule junctions [10–16] is the close match of their Fermi energy with the highest occupied molecular orbital (HOMO) or lowest unoccupied molecular orbital (LUMO) energy levels of organic molecules. Furthermore, in comparison to the more bulky metallic electrodes, graphene electrodes promote electrostatic gating as a result of the reduced screening.

Here, we study the charge and thermal transport characteristics through a porphyrin single-molecule transistor with electro-burnt graphene electrodes using the nonequilibrium Green’s function method and density functional theory. First we discuss the electronic structure of the prophyrin molecule. Then we study the electro-burnt graphene electrodes and finally the two-terminal device in which the anchor groups of the porphyrin molecule bind to the graphene electrodes by p-orbital overlap. We then discuss the contribution of each part of the molecule to charge transport by means of an effective tight binding model. Finally, we investigate the thermoelectric properties of the device.

## Results and Discussion

**Figure 1:**
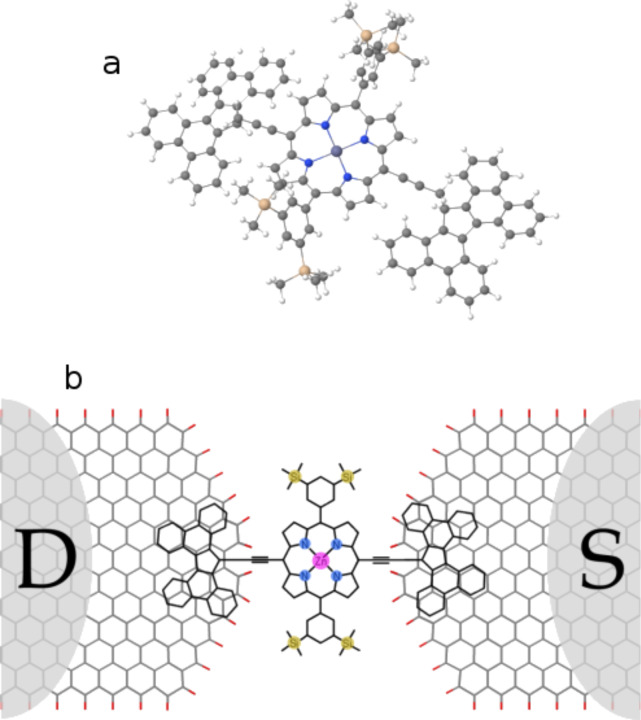
Porphyrin SET molecular structure. a) Porphyrin molecule with butterfly backbone, b) the device structure consisting of two electro-burnt graphene electrodes, a porphyrin molecule and reservoirs D and S. The butterfly anchor groups of the porphyrin molecule are connected to the surface of the graphene through π–π interactions, resulting in mixed AA and AB stacking with graphene.

Figure 1 shows the molecular structure of the porphyrin-based single-molecule transistor (SET), which consists of two electro-burnt graphene electrodes connected to drain and source reservoirs (D and S) and the porphyrin molecule (PM). The molecule consists of two “butterfly” anchor groups connected to the porphyrin core via a carbon acetylene spacer, as shown in Figure 1a. The anchor groups bind to the surface of the graphene through π–π interactions. The central porphyrin is also connected to two side groups, which stabilize the molecule within the junction.

We first use density functional theory (DFT) to study the electronic structure of the PM. To characterize the gas phase molecule, the isolated PM shown in Figure 1a is relaxed to reach the ground state energy (see Computational Methods). We carried out a spin-polarized calculation since the d orbitals of the Zn atom could be filled to different degrees. It is well-known that Kohn–Sham DFT eigenvalues usually underestimate the HOMO–LUMO gap and DFT typically does not predict their correct location relative to the Fermi energy of the electrodes. However, the LUMO (HOMO) energy level is almost equivalent to the negative of the electron affinity energy (ionization potential). Therefore, we estimate the bandgap, *E*_g_, of the molecule (sometimes called additional energy) by computing the electron affinity (*EA*) and ionization potential (*IP*): *E*_g_ = *IP* – *EA*. The *EA* and *IP* are calculated from the total energy of the neutral and *N* ± 1 electron states of the molecule: *IP* = *E*(*N*−1) − *E*(*N*), and *EA* = *E*(*N*) − *E*(*N*+1). For the PM, the *IP* and *EA* are calculated as 5.2 eV and 1.36 eV, respectively, which yields *E*_g_ = 3.84 eV. The Kohn–Sham DFT eigenvalues predict 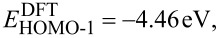 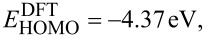 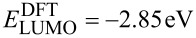
and 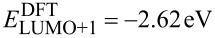
using the generalised gradient approximation/Perdew–Burke–Ernzerhof exchange-correlation functional (GGA/PBE), which results in a Kohn–Sham DFT gap of 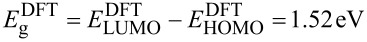
for the gas phase molecule.

**Figure 2:**
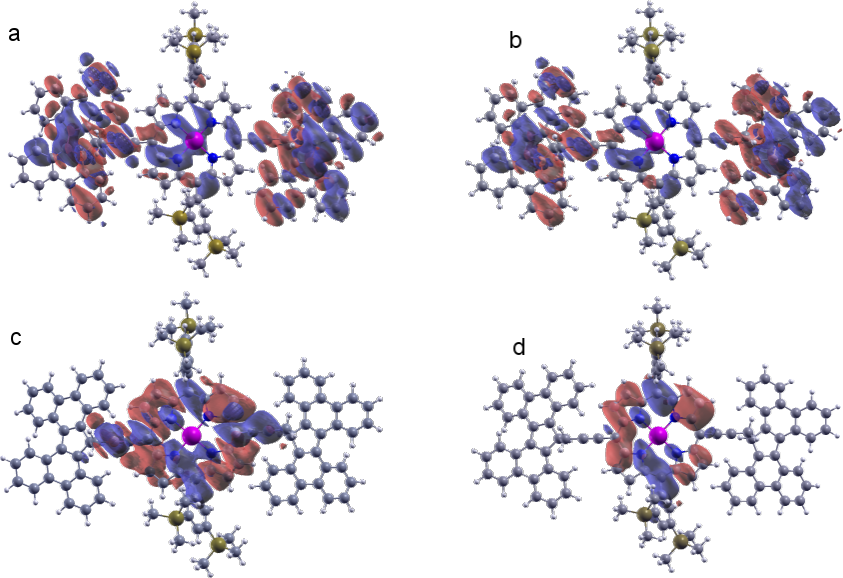
a) HOMO−1, b) HOMO, c) LUMO and d) LUMO+1 state iso-surfaces.

Figure 2 shows the iso-surfaces of the HOMO−1, HOMO, LUMO and LUMO+1 states. The wave function of the HOMO−1 and HOMO states are mostly delocalized over the PM and the butterfly anchors, whereas the LUMO and LUMO+1 states are localized on the porphyrin central group. This suggests transport should be HOMO dominated. However, the molecular orbitals are localized in the porphyrin central group in the LUMO and LUMO+1 state.

### Electro-burnt graphene electrodes

Feedback-controlled electro-burnt graphene (EBG) electrodes with nanometre separation were formed using mechanically exfoliated, few-layer graphene [9] and CVD-grown, monolayer graphene [17–18]. To form the EBG electrodes, first a sufficiently high bias voltage is applied to create cracks in the naoribbon. This usually happens in the centre of the nanoribbon due to the higher temperature induced in this region [19]. This high temperature in the constricted part of the graphene nanoribbon causes the carbon atoms to instantaneously react with atmospheric oxygen, resulting in combustion. A feedback signal is used to impede this oxidation before the sample is destroyed. After successive repetitions of this process, the graphene nanoribbon becomes more and more narrow and finally breaks to create a nanometre-sized gap. The molecule can be placed in this gap, enabling the study of its electrical properties. Moreover, the ability to place a gate electrode beneath the gap makes this an excellent platform for tuning and studying quantum effects in single-molecule transport.

**Figure 3:**
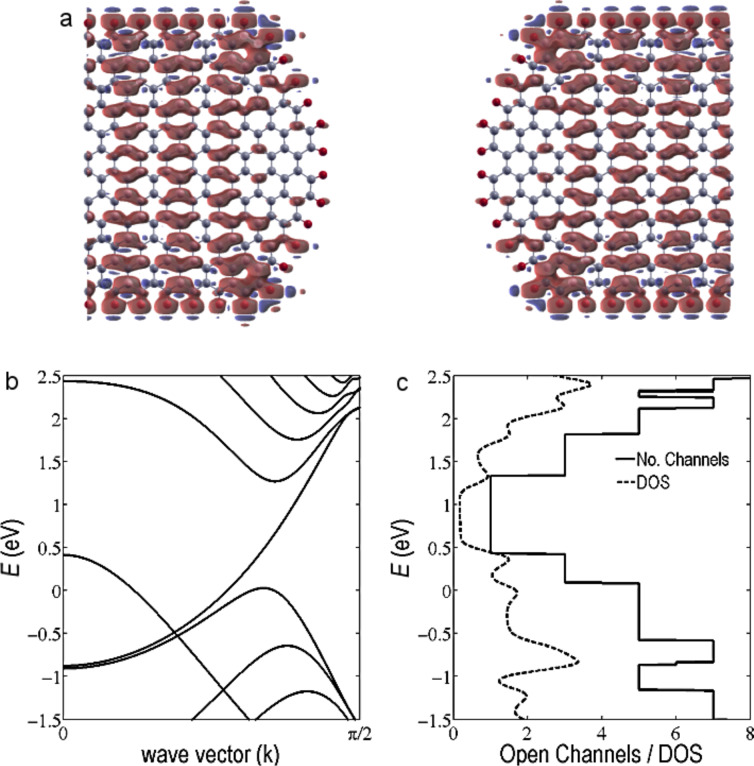
Molecular and electronic structure of the EBG electrodes. a) Molecular orbital iso-surfaces, b) band structure of EBG electrodes, c) density of states and number of open channels in the EBG electrodes.

Due to the combustion process, the edges of the EBG are likely terminated by oxygen, especially close to the junction. Therefore, before studying the transport properties of the PM, we focus on the transport properties of the EBG electrodes with oxygen-terminated edges. Figure 3 shows the molecular and electronic structure of the EBG electrode. The electrode is a 3 nm wide, zigzag, semi-infinite, graphene nanoribbon terminated by oxygen and connected to a half-ellipse-like structure as shown in Figure 3a. The molecular orbital levels in the Fermi energy, *E*_F_ = 0 eV, indicate that the orbitals are mostly localized in the edges of the ribbon. It is apparent that the up-spin is mostly located toward the edges in contrast with the down-spins, which are delocalized over the EBG. The band structure of the electrode is bent in the vicinity of the k point due to the edge states associated with the oxygen atoms (Figure 3b). Due to the high electronegativity of oxygen atoms, charge is transferred from the carbon atoms to the oxygen atoms. Consequently, oxygen-terminated ribbons show p-type doped behaviour [20–21] and their density of states (DOS) is shifted toward positive energies as shown in Figure 3c (dashed line). Figure 3c (solid line) also shows the number of open conduction channels in ideal-oxygen-terminated EBG electrodes. Due to the p-type behaviour of EBG electrodes, the open channels are shifted toward the positive energies.

### Electron and thermal transport in EBG–PM junction

**Figure 4:**
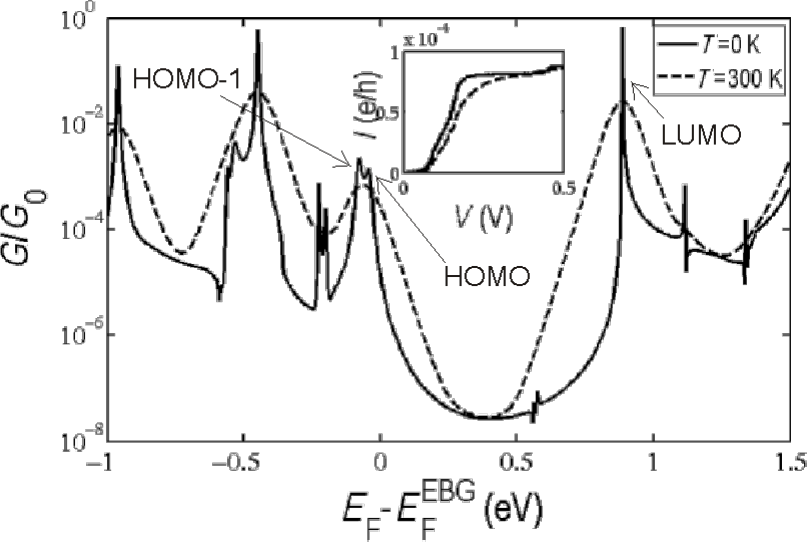
Conductance, *G*/*G*_0_, as a function of the Fermi energy, *E*_F_, of the EBG electrodes shown in Figure 1b.

We now consider transport through the molecule inside the nanogap. In the equilibrium configuration, the PM butterfly anchor groups assume a mixed AA and AB stacking with the graphene surface. Figure 4 shows the resulting transmission probability for the electrons of energy *E* passing from the left to the right electrode at 0 K and 300 K, obtained from the DFT mean-field Hamiltonian of the system combined with our Green’s function (*GF*) code (see Computational Methods). Due to the presence of oxygen atoms in the edges of the EBG electrodes, the Fermi energy is predicted to shift by about 0.45 eV, compared with hydrogen-terminated electrodes and bare EBG electrodes. The HOMO−1, HOMO and LUMO resonances are labelled in Figure 4. As discussed above, the DFT-predicted Kohn–Sham eigenvalues for the HOMO and HOMO−1 states are quite close to each other, which is why the HOMO and HOMO−1 resonances overlap. Moreover, the Fermi energy is close to the HOMO resonance, indicating HOMO-dominated transport, which is consistent with the local density calculation shown in Figure 2 for a gas phase molecule. The inset of Figure 4 shows the zero-bias current of the PM–EBG device at 0 K and 300 K.

**Figure 5:**
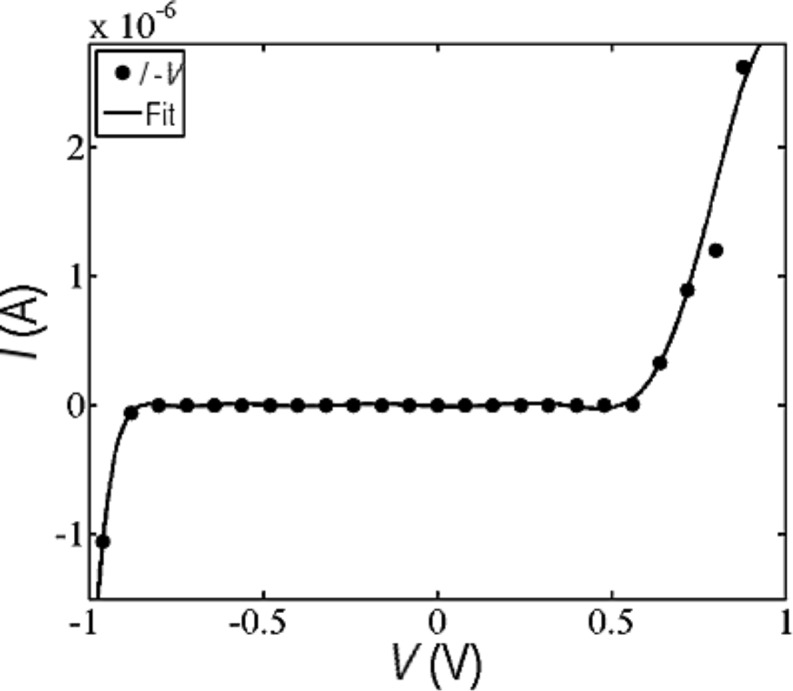
Nonequilibrium *I*–*V* characteristic of the PM–EBG device.

Under nonequilibrium conditions at finite voltages, the transmission coefficient, *T*(*E*,*V*), depends on both the electron energy, *E*, and bias voltage, *V*, which in the presence of asymmetries can lead to rectification in the PM–EBG device. To demonstrate this, we have calculated the *I*–*V* characteristic of the device using the voltage-dependent transmission coefficient, as shown Figure 5, for voltages in [−1,1] V, which is an experimentally applicable voltage range for this device.

Remarkably the current is not symmetric relative to the zero-bias point, even though the molecule itself and the leads are ideally symmetric. The asymmetry is due to differences in the angle between the PM butterfly anchor and the spacer between the prophryin and anchor, which results in a slightly different configuration in the left and right electrodes. As a consequence, the junction configuration and coupling to the electrodes are slightly asymmetric, leading to rectification. Moreover, for a wide bias window [−0.9,0.6] V, the current is almost zero, but then suddenly increases with a high current flow capability in the range of microamperes*.*

**Figure 6:**
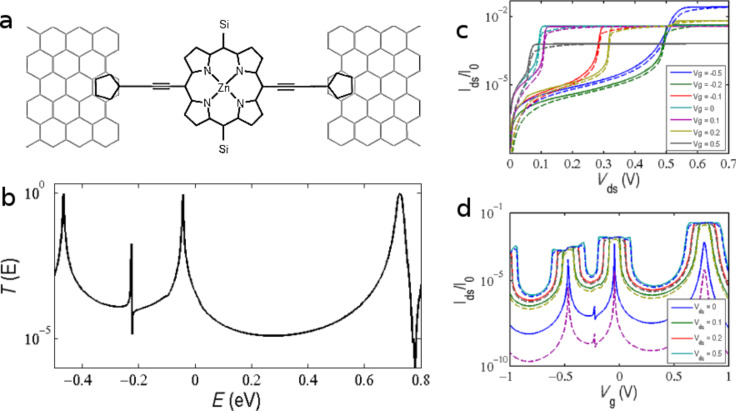
Tight-binding model of the PM–EBG device. (a) Schematic of the simplified device for tight-binding calculation, (b) transmission coefficient *T*(*E*) using Hückel parameters, (c) drain source current (*I*_ds_) as a function of the drain source (bias) voltage (*V*_ds_) and (d) drain source current as a function of the gate voltage (*V*_g_).

To further investigate transport through the device, we constructed a tight-binding model of the simplified device (Figure 6a) using modified Hückel parameters (see Computational Methods). The model is in good agreement with the *T*(*E*) calculated from the DFT mean-field Hamiltonian as shown in Figure 6b. A Fano resonance appearing close to *E* = −0.2 eV is associated with the nitrogen atoms. By changing the coupling of the N–C, their position changes, and interesting features can result close to the HOMO that could be used in thermoelectric devices. Furthermore, they could be split if slightly asymmetric coupling between carbon and nitrogen is applied. Neither the Zn nor the side groups (represented in Figure 6a by “Si”) have an effect on the electron transport since they have high orbital energies far from the HOMO–LUMO resonances. Although the current mostly passes through the edge of the ribbon, it does not have much effect on the transport due to the weak coupling between the anchor and electrode surfaces.

We used the tight-binding (TB) model to investigate the behaviour of the PM–EBG device (Figure 1b) with a perpendicular gate voltage applied. This is modelled by calculating the gate voltage dependence of the transmission coefficient, *T*(*E*,*V*_g_). To obtain a *V*_g_-dependent TB Hamiltonian, a gate potential is added to the diagonal terms of the TB Hamiltonian. Obviously this does not take the Coulomb energy effect into account. Figure 6c shows the drain–source current (*I*_ds_) as a function of the drain–source (bias) voltage (*V*_ds_) at 0 K (solid lines) and room temperature (dashed lines). When *V*_g_ = 0.5 V is applied, the *I*_ds_ switches to the on-state much faster and saturates as compared to *V_g_* = 0 or −0.5 V, as shown in Figure 6c. The on/off state of the device does not change monotonically with the gate voltage, which could be explained by the asymmetry of the transmission coefficient around the theoretical Fermi energy (*E* = 0). However, in the [−0.1,0.1] V interval, the current could be changed from ≈2 × 10^−5^ to ≈3 × 10^−3^, which gives the ratio of 150. The high variation of the current in a small gate voltage window shows the potential of the device to perform in digital electronics. Figure 6d shows the drain source current (*I*_ds_) as a function of the gate voltage (*V*_g_) for different bias voltages. When the bias voltage is very small (*V*_ds_ ≈ *0*), the *I*_ds_−*V*_g_ dependence is quite similar to the *T*(*E*). As expected, at high bias voltages exceeding the width of the transmission resonance, the *I*_ds_ remains constant over a range of *V*_g_ of order *V*_ds_.

**Figure 7:**
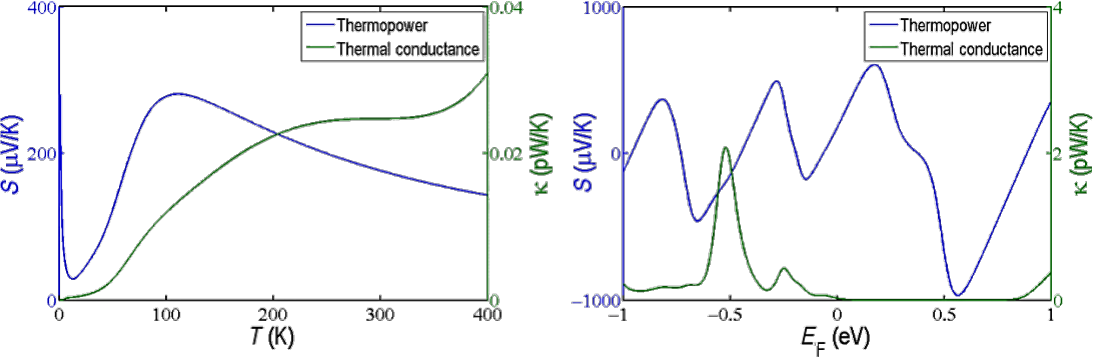
Thermal properties of the PM–EBG device. Thermopower (blue) and thermal conductance (green) as a function of the (a) temperature at *E*_F_*=* 0 eV and (b) Fermi energy *E*_F_ at room temperature.

Figure 7 shows the thermopower, *S*, and the electronic contribution to the thermal conductance, κ, of the PM–EBG device. The device generated a maximum thermopower of 280 μV/K at 110 K, which then decreased at higher temperatures, as shown in Figure 7a. Furthermore, the thermal conductance of the device increases with temperature to values on the order of 0.025 pW/K at room temperature. In addition, as shown in Figure 7b, the thermopower changes sign for different Fermi energies (*E*_F_) because the Fermi energy is located close to a resonance. Consequently, a variation in the Fermi energy as small as 10 meV increases the thermopower to 475 μV/K. In contrast, the electronic thermal conductance of the device is quite low and does not change significantly with the small variation of the Fermi energy. The thermoelectric figure-of-merit could be high in this device provided the phonon contribution to the thermal conductance is small compared to the electronic contribution [22]. Therefore, the PM–EBG device shows great potential as a thermoelectric device.

## Conclusion

We have investigated the electrical and thermoelectrical properties of a porphyrin-based molecule bound to electro-burnt graphene electrodes by planar aromatic anchor groups. Due to the efficient π–π overlap between the anchor groups and graphene and the location of frontier orbitals relative to the graphene Fermi energy, we predicted HOMO-dominated transport and a positive thermopower as high as 280 μV/K. By gating the junction and tuning the Fermi energy, this can be further enhanced. Although the electrodes and molecule are symmetric, the junction itself can be asymmetric, due to different binding configurations at the electrodes. This can lead to slight rectification in the current–voltage characteristic of the junction.

## Computational Methods

The Hamiltonian of the structures described in this paper were obtained using density functional theory (DFT) as described below or constructed from a simple tight-binding model (TB), as shown in Figure 6a. The single orbital per atom of site energies are ε_C_ = −4.05, ε_H_ = −20.5, ε_Si_ = −6.03, ε_N_ = −4.66, ε_Zn_ = −20.7, and nearest neighbour couplings γ_C–C_ = −2.7, γ_C–H_ = −0.23, γ_C–N_ = −4.2, γ_C–Si_ = −3.51 and γ_N–Zn_ = −2.9 and couplings to the right and left electrodes γ_RS_ = −0.27 and γ_LS_ = −0.35, respectively.

**DFT calculation:** In a similar manner as described in [23], the optimized geometry and ground state Hamiltonian and overlap matrix elements of each structure was self-consistently obtained using the SIESTA [24] implementation of density functional theory. SIESTA employs norm-conserving pseudo-potentials to account for the core electrons and linear combinations of atomic orbitals to construct the valence states. The generalized gradient approximation (GGA) of the exchange and correlation functional is used with the Perdew–Burke–Ernzerhof parameterization (PBE) [25], a double-ζ-polarized (DZP) basis set, and a real-space grid defined with an equivalent energy cut-off of 150 Ry. The geometry optimization for each structure was performed for forces less than 200 meV/Å. The local density of state calculation was performed with a polarized configuration and at zero (electronic) temperature. For the band structure calculation, the EBG electrode was sampled by a 1 × 1 × 500 Monkhorst–Pack k*-*point grid.

**Transport calculation*:*** In a similar manner as described in [26–27], the mean-field Hamiltonian obtained from the converged DFT calculation or a simple tight-binding Hamiltonian was combined with our implementation of the nonequilibrium Green’s function method, the GOLLUM [28], to calculate the phase-coherent, elastic scattering properties of each system consisting of left (source) and right (drain) electrodes and the scattering region. The transmission coefficient, *T*(*E*), for electrons of energy *E* (passing from the source to the drain) is calculated via the relation:

[1]
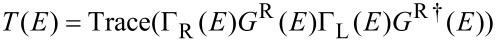


In this expression, 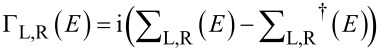 describes the level broadening due to the coupling between left (L) and right (R) electrodes and the central scattering region (S). The sum 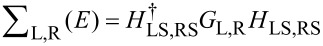 represents the retarded self-energies associated with this coupling. *H*_LS,RS_ and *G*_L,R_ are the coupling matrices between LS and RS and the surface Green’s function of the electrodes, respectively. *G*^R^ = (*ES*−*H*_S_−Σ_L_−Σ_R_)^−1^ is the retarded Green’s function, where *H*_S_ is the Hamiltonian of the scattering region and *S* is the overlap matrix.

**Electrical properties:** In a similar manner as described in [23], using the obtained transmission coefficient, *T*(E), the conductance could be calculated by the Landauer formula, 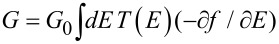, where *G*_0_ = 2e^2^/h is the conductance quantum. In addition, the zero-bias current through the device at voltage *V* could be calculated as:

[2]
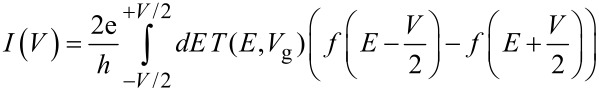


where *f*(*E*)=(1+exp((*E*−*E*_F_)/*k*_B_*T*))^−1^ is the Fermi–Dirac distribution function, *T* is the temperature, *V*_g_ is the gate voltage and *k*_B_ = 8.6 × 10^−5^ eV/K is Boltzmann’s constant. Under nonequilibrium conditions, the *I*_ds_–*V*_ds_ characteristic could be calculated from the voltage-dependent transmission, *T*(*E*,*V*_ds_), as

[3]
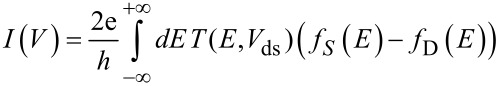


where the *f*_S,D_ are the electrochemical potential in the drain and source.

**Thermal properties:** In a similar manner as described in [29–30], the thermal properties of a given device could be calculated from the transmission coefficient, *T*(*E*). The thermopower (*S*) and electronic contribution in thermal conductance (κ_e_) as a function of the temperature (*T*) can be written as:

[4]
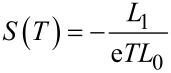


[5]
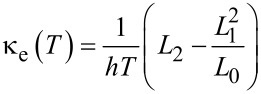


where 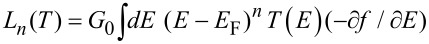.
